# Structural and Functional Interrogation of Active *Streptococcus pneumoniae* Sortase A

**DOI:** 10.3390/biom16091231

**Published:** 2026-08-25

**Authors:** Eunjeong Lee, Blaine Hunter Gordon, Jasmina S. Redzic, Anthony J. Saviola, Sean P. Maroney, Steven Shaw, Mila Cordero, Shaun Bevers, Angelo D’Alessandro, Kirk C. Hansen, Sarah E. Clark, Elan Eisenmesser

**Affiliations:** 1 Department of Biochemistry and Molecular Genetics, School of Medicine, University of Colorado Anschutz Medical Campus, Aurora, CO 80045, USA; eunjeong.lee@cuanschutz.edu (E.L.); jasmina.redzic@ucdenver.edu (J.S.R.); anthony.saviola@cuanschutz.edu (A.J.S.); sean.maroney@ucdenver.edu (S.P.M.); milacordero111@gmail.com (M.C.); angelo.dalessandro@cuanschutz.edu (A.D.); kirk.hansen@cuanschutz.edu (K.C.H.); 2 Biopharmaceutical Research Center, Korea Basic Science Institute, 162 Yeongudanji-Ro, OchangEup, Cheongju-Si 28119, Republic of Korea; 3 Department of Otolaryngology—Head & Neck Surgery, School of Medicine, University of Colorado Anschutz Medical Campus, Aurora, CO 80045, USA; steven.c.shaw@cuanschutz.edu (S.S.); sarah.e.clark@cuanschutz.edu (S.E.C.)

**Keywords:** Sortase A, *Streptococcus pneumoniae*, NMR, enzyme

## Abstract

Sortase A (SrtA) enzymes covalently anchor surface proteins to Gram-positive bacterial cell walls, promoting colonization and virulence. In *Streptococcus pneumoniae*, previous studies identified both a domain-swapped dimer and an active refolded monomer, but the active enzyme has not been characterized at the structural and residue-specific level. Here, we performed quantitative proteomic comparisons of wild-type and SrtA knockout strains that confirmed the loss of multiple LPxTG-containing virulence factors, including ZmpB, NanA, and IgA1 protease, consistent with an essential role for SrtA in surface protein anchoring. To enable mechanistic studies, we established a biochemical framework to produce monomeric *Streptococcus pneumoniae* SrtA by refolding and developed a gel-based assay using recombinant substrates to monitor catalytic activity. The refolded monomer, but not the swapped dimer, catalyzed cleavage and transpeptidation of a canonical LPxTG substrate in a metal-independent manner under the conditions examined. We further report high-resolution NMR backbone assignments for the active monomer and identify substrate-induced chemical shift perturbations that localize to the active site. Together, these findings provide an integrated proteomic, biochemical, and NMR characterization of monomeric, catalytically active *Streptococcus pneumoniae* SrtA and reveal residue-specific interactions with a canonical LPNTG recognition peptide.

## 1. Introduction

*Streptococcus pneumoniae* (*S. pneumoniae*) remains a leading cause of morbidity and mortality worldwide and is now classified by the World Health Organization as a “high-priority” pathogen due to rising antimicrobial resistance and the limited coverage of current glycoconjugate vaccines [1,2]. With over 90 known serotypes, and non-vaccine serotypes increasingly contributing to disease, there is a pressing need to identify protein-based targets for both therapeutic and vaccine strategies [3,4].

**Figure 1:**
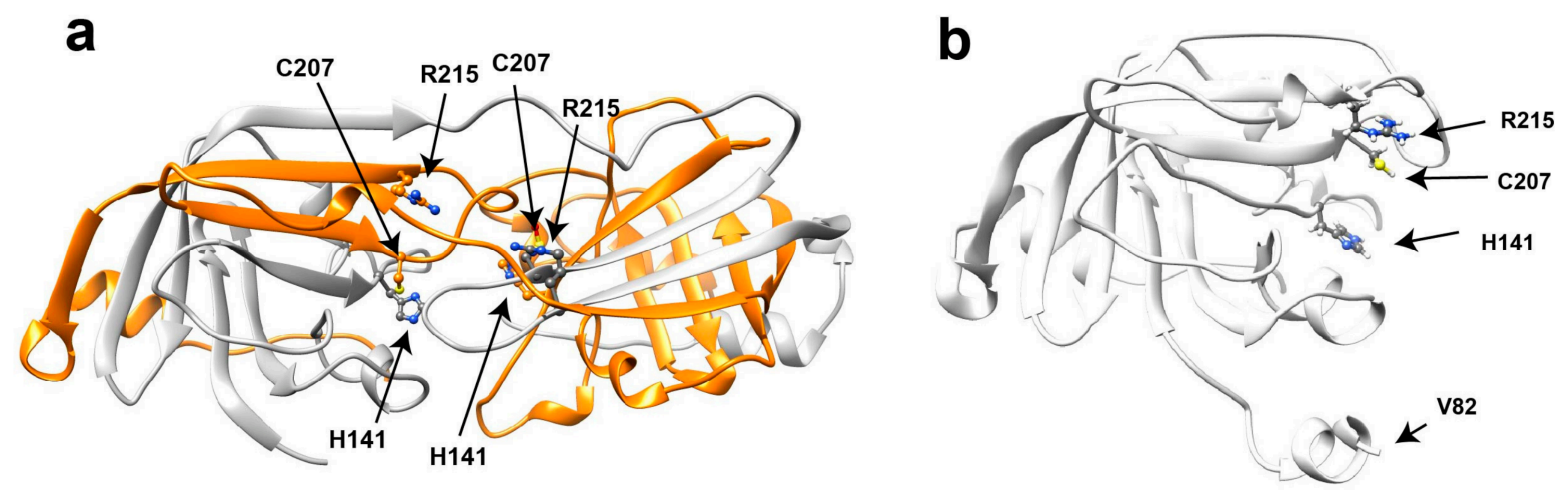
*S. pneumoniae* SrtA forms a domain-swapped dimer. (**a**) The previously determined X-ray crystal structure of the SrtA domain-swapped dimer (PDB accession 4O8L). One monomer is colored white and the other is colored orange. (**b**) The AlphaFold predicted structure of the SrtA monomer. The catalytic triad of H141-C207-R215 are delineated as ball-and-stick in both models (arrows).

One such promising target is the sortase A (SrtA) enzyme—a highly conserved, “housekeeping” transpeptidase responsible for anchoring dozens of surface proteins to the bacterial cell wall by recognizing LPxTG sorting signals and catalyzing their covalent linkage to the peptidoglycan [5,6]. These substrates include virulence factors critical for biofilm formation, adherence, and invasion [7]. For example, the presence of SrtA is essential for epithelial adherence and its deletion impairs nasopharyngeal colonization in multiple animal models [8,9]. The first crystal structure of *S. pneumoniae* SrtA revealed an unusual domain-swapped dimer with substantially reduced catalytic activity (Figure 1) [10]. This domain-swapped architecture contrasts with the well-characterized *Staphylococcus aureus* (*S. aureus*) SrtA, which does not undergo domain swapping but instead relies on calcium to facilitate catalysis [6]. Subsequent biochemical studies demonstrated that recombinant *S. pneumoniae* SrtA could be refolded into an active monomer and further established that catalysis is calcium-independent [11]. Nevertheless, the structural properties of the active monomer and its residue-specific interactions with the substrate have remained unexplored.

SrtA enzymes catalyze transpeptidation through a conserved two-step mechanism mediated by a catalytic triad of histidine, cysteine, and arginine [6]. The reaction is initiated when the thiol group of the active site cysteine performs a nucleophilic attack on the carbonyl carbon of the threonine within the LPxTG sorting motif, forming a covalent thioacyl-enzyme intermediate. This intermediate is subsequently resolved by nucleophilic attack from an amino group on a peptidoglycan precursor, completing the covalent attachment of the substrate to the cell wall. The histidine residue functions as a general base, activating the cysteine for nucleophilic attack, while the arginine stabilizes the negative charge that develops in the transition state. In *S. pneumoniae* SrtA, this triad is composed of His141, Cys207, and Arg215. However, mutational and mechanistic studies indicate that the contribution of the conserved arginine to catalysis is more complex and may not be essential for all steps of the reaction [12]. Although refolded monomeric *S. pneumoniae* SrtA has been shown to be catalytically active [11], the structural properties of the active enzyme and its residue-specific interactions with LPxTG-containing substrates remain unexplored.

Here, we complement previous biochemical studies of *S. pneumoniae* SrtA by combining quantitative proteomics, biochemical assays, and solution NMR spectroscopy. Quantitative proteomic profiling of a Δ*srtA* strain confirms the loss of multiple LPxTG-containing surface proteins, consistent with the central role of SrtA in surface protein anchoring. Using refolded monomeric SrtA, we further characterize its catalytic properties, demonstrate metal-independent cleavage and transpeptidation of canonical LPxTG substrates, and identify substrate-induced chemical shift perturbations that localize to the active site. Together, these findings provide an integrated proteomic, biochemical, and NMR characterization of catalytically active *S. pneumoniae* SrtA and reveal residue-specific interactions with a canonical LPNTG recognition peptide.

## 2. Materials and Methods

### 2.1. Bacterial Strains and Growth Conditions

Wild-type (WT) *S. pneumoniae* serotype 2 strain D39 [13] and the ∆*srtA* knockout strain were cultured in Todd-Hewitt broth supplemented with 0.5% yeast extract (THY) or on blood agar plates at 37 °C in 5% CO_2_. For antibiotic selection, ∆*srtA* strains were grown in THY supplemented with kanamycin (300 μg/mL).

The ∆*srtA* strain was constructed by replacement of the complete *srtA* coding sequence (SPD_1076; genomic coordinates 1,102,252–1,102,995 in *S. pneumoniae* D39) with the Sweet Janus cassette using homologous recombination [14]. The replacement construct contained the complete Sweet Janus cassette flanked by 1001 bp regions immediately upstream and downstream of *srtA*. The resulting construct was introduced into *S. pneumoniae* by transformation in minimal medium containing competence-stimulating peptide and verified by PCR. Transformants were selected on blood agar plates containing kanamycin (300 μg/mL). Replacement of *srtA* by the Sweet Janus cassette was confirmed by colony PCR and DNA sequencing.

### 2.2. Proteomic Analysis of SrtA-Dependent Expression Changes

To examine proteomic differences associated with *S. pneumoniae* SrtA function, WT and ∆*srtA* strains were grown in THY broth to an optical density (OD_600_) of ~1.0. Bacteria were harvested by centrifugation at 5000× *g* for 10 min at 4 °C, washed twice in PBS, and processed for MS analysis using S-Trap™ micro spin columns (Protifi, Fairport, NY, USA) following the manufacturer’s protocol.

Protein digests were loaded onto Evotips and separated using an Evosep One LC system with a PepSep column (150 μm ID × 15 cm, Bruker, Marslev, Denmark, packed with ReproSil-Pur C18 1.9 μm resin). Eluted peptides were introduced into a timsTOF Pro mass spectrometer (Bruker, Billerica, MA, USA) equipped with a CaptiveSpray source and operated in PASEF mode. Ten MS/MS scans per topN cycle were acquired with a 100 ms ramp time. Spectra were collected across a mass range of *m*/*z* 100–1700, with ion mobility scanned from 0.7 to 1.5 Vs/cm^2^. Precursors were isolated within ±1 Th and fragmented with mobility-dependent collision energy (20–59 eV). Low-abundance precursors (intensity >500 but <20,000 counts) were permitted to reacquire unless dynamically excluded (0.4 min).

Data were processed using FragPipe (v19) and searched with MSFragger against the *S. pneumoniae* D39 proteome. Peptide-spectrum matches were filtered to 1% FDR using Percolator, and protein quantification was performed using IonQuant. Differential protein abundance was analyzed using MetaboAnalyst v5.0 using a two-sample *t*-test to compare WT and Δ*srtA* strains. Proteins exhibiting |log2FC| > 1 and *p* < 0.05 were considered significantly altered. The complete protein-level datasets underlying these analyses, including fold changes and raw *p*-values, are provided in Supplementary Data S1 and S2. FDR correction was additionally applied to the statistical analyses, with the corresponding FDR-corrected datasets provided in Supplementary Data S3 and S4.

In addition, to assess the presence of covalent SrtA reaction products, Supplementary Data S5 provides mass spectrometry results from the gel-excised thioacyl-intermediate band confirming identification of both the SUMO-LPNTG and SrtA components.

### 2.3. Cloning and Purification

All recombinant constructs were ordered from Integrated DNA Technologies (Coralville, IA, USA) and cloned into a pET21 vector. For SrtA enzymes, an N-terminal 6xHis tag was encoded for both *S. pneumoniae* SrtA (Uniprot A0A0H2ZM58, residues 82–247) and *S. aureus* SrtA (Uniprot Q2FV99, residues 60–206). These constructs comprise the soluble catalytic domains of the respective enzymes and omit their N-terminal membrane-anchoring regions. For substrate constructs, an N-terminal human SUMO was fused at its C-terminus to either: (1) a 20-residue peptide containing the LPNTG recognition motif (NQLAELPNTGSKNERQALYS; “SUMO-LPNTG”) or (2) the Streptococcal protein G B1 domain with an N-terminal glycine tetrapeptide (G_4_-GB1). Both SUMO constructs also contained an N-terminal 6xHis tag.

For recombinant protein expression, constructs were transformed into *E. coli* BL21(DE3) cells and selected with ampicillin. Cultures were grown in 4 L LB medium, induced with isopropyl-β-D-thiogalactopyranoside (IPTG) at 0.6–0.8 OD_600_ for 3 h at 37 °C, harvested by centrifugation, and frozen until purification.

For purification of unlabeled SrtA, we purified SrtA denatured and applied a refolding protocol as described previously [15,16,17]. Briefly, cells were lysed by sonication in 5 M guanidine-containing Ni buffer (50 mM Na_2_PO_4_, pH 7.0, 500 mM NaCl, 10 mM imidazole), clarified by centrifugation, and applied to Ni-affinity resin (Sigma-Aldrich, St. Louis, MO, USA). Bound protein was eluted with Ni B (1 M imidazole) in 5 M guanidine and 30–40 mL of the elutions were refolded via dialysis into refold buffer (50 mM Tris, pH 7.5, 100 mM NaCl, 1 M arginine, 1 mM DTT) for at least 12 h. Dialysis was continued into NMR buffer (50 mM HEPES, pH 7.0, 150 mM NaCl, 1 mM DTT), and the refolded material was concentrated and further purified by size-exclusion chromatography (SEC) on a Sephadex-75 preparative column (Cytiva, Marlborough, MA, USA). Fractions containing monomeric active enzyme were pooled and stored at –80 °C.

For SUMO-fusion substrates, soluble lysates were applied to Ni-affinity columns, eluted with Ni B, and dialyzed back into Ni A. The 6xHis-SUMO tag was cleaved using ULP1 (produced in-house from Addgene plasmid pFGET19_ULP1), and the cleaved reaction passed again through Ni resin to remove tag and protease. Flow-through containing the substrate was concentrated and purified by SEC in NMR buffer, then flash frozen for storage.

For isotopically labeled proteins, cultures were grown in 4 L LB to 0.5 OD_600_, pelleted, and resuspended in 1 L minimal media. Following a 20 min adaptation period, antibiotics were re-added and IPTG was added after an additional 20 min. Standard M9 minimal media was used with ^15^N-NH_4_Cl (Sigma, St. Louis, MO, USA) or both ^15^N-NH_4_Cl and ^13^C-glucose (Sigma) for double-labeled samples.

### 2.4. Catalytic Assays

Catalytic activity of SrtA enzymes was assessed using both gel-based and real-time FRET-based assays.

For the gel-based transpeptidation assay, 250 μM SrtA was incubated with 500 μM SUMO-LPNTG (acyl donor) and 500 μM G_4_-GB1 (nucleophile) in 50 mM Tris, 150 mM NaCl, pH 7.5. Reactions were incubated overnight at 25 °C. Reaction products were analyzed by SDS-PAGE and visualized by Coomassie staining. Formation of the expected ligation product (SUMO-LPNT–G_4_-GB1) and intermediate species was confirmed by mass spectrometry.

To independently monitor *S. pneumoniae* SrtA cleavage activity, a FRET-based internally quenched peptide substrate containing an LPETG motif was used (Anaspec, Fremont, CA, USA). Specifically, this substrate is Abz-LPETG-K(Dnp)-NH2. All measurements were collected on a BioTek Synergy H1 microplate reader (Agilent Technologies, Santa Clara, CA, USA). Reactions were performed using 10 μM SrtA and 5–60 μM substrate in the same buffer. Fluorescence was monitored at 25 °C (Ex: 340 nm; Em: 490 nm). Product formation was quantified using a standard curve generated from full hydrolysis of the FRET peptide incubated overnight with 1 mg/mL proteinase K (Promega Corporation, Madison, WI, USA) at 37 °C. Initial velocities were calculated from the linear portion of the fluorescence trace and reported as mol·s^−1^. Data were fit using GraphPad Prism version 10 software (GraphPad Software, San Diego, CA, USA).

## 3. Results

### 3.1. Loss of SrtA Selectively Depletes LPxTG-Containing Surface Proteins

**Figure 2:**
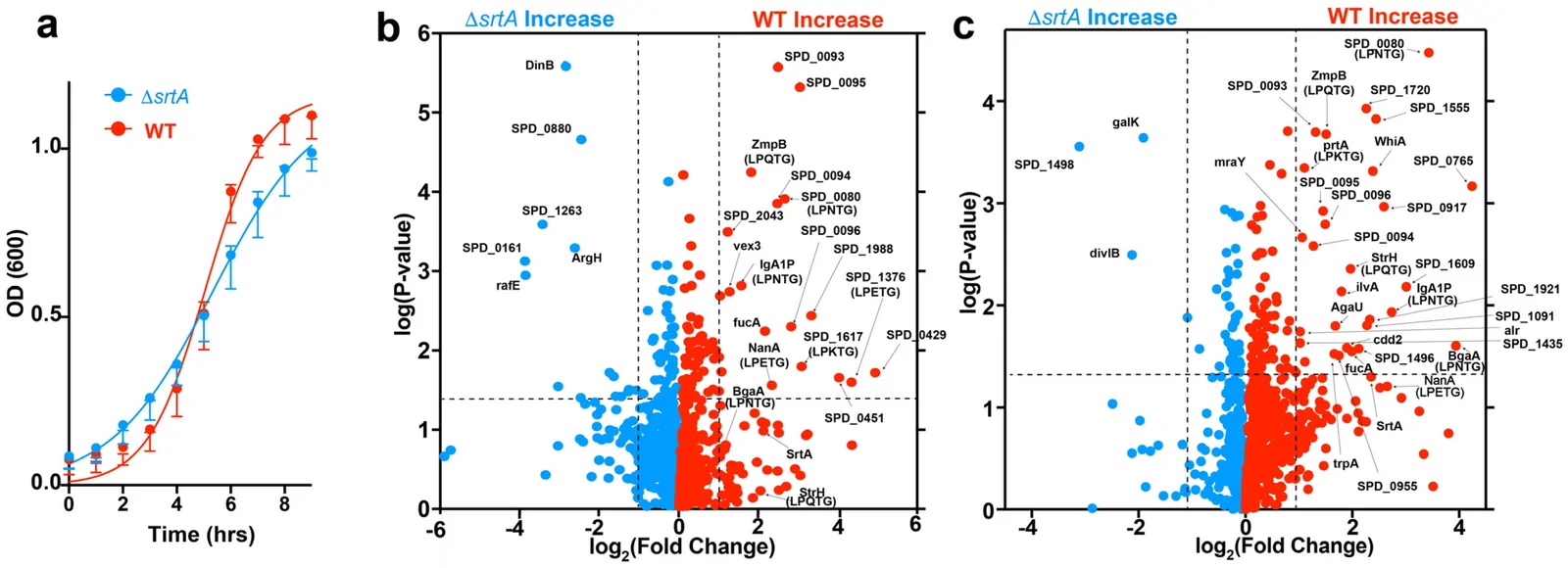
Deletion of *srtA* alters growth kinetics and the surface proteome of *S. pneumoniae*. (**a**) Growth curves of WT D39 and ∆*srtA* strains cultured under the same conditions used for quantitative proteomic analysis. The ∆*srtA* strain exhibited delayed growth but reached a similar final saturated cell density as the WT strain (OD600 ≈ 1.0). Data represent the mean ± SD of three measured replicates. Curves were fit using a logistic growth model, yielding R^2^ values of 0.989 and 0.983 for WT and ∆*srtA*, respectively. (**b**) Volcano plot from the first quantitative proteomic analysis comparing surface-associated proteins from WT D39 and the Δ*srtA* strain. Each condition comprised three biological replicates. Proteins significantly enriched in the Δ*srtA* strain are shown in blue, whereas proteins significantly depleted in the Δ*srtA* strain are shown in red. Canonical LPxTG-containing SrtA substrates are labeled. Complete protein-level data and the corresponding FDR-corrected analysis are provided in Supplementary Data S1 and S3, respectively. (**c**) Biological replicate of the quantitative proteomic analysis shown in (**b**). Complete protein-level data and the corresponding FDR-corrected analysis are provided in Supplementary Data S2 and S4, respectively.

We first compared the growth of WT D39 and the isogenic Δ*srtA* strain under the same conditions used for proteomic analysis. The Δ*srtA* strain exhibited delayed growth relative to the WT strain (Figure 2a). Logistic fitting of the growth curves yielded an apparent growth rate constant (*k*) of 0.53 for the Δ*srtA* strain compared with 0.93 for the WT strain, although both strains ultimately reached similar final cell densities. We next compared the surface-associated proteomes of WT and Δ*srtA* bacteria using quantitative mass spectrometry in two independent biological experiments, each comprising triplicate cultures. The complete protein-level datasets underlying these analyses, including fold changes and raw *p*-values, are provided in Supplementary Data S1 and S2, with the corresponding FDR-corrected analyses provided in Supplementary Data S3 and S4

Analysis of both proteomic datasets revealed relatively few proteins consistently enriched in the Δ*srtA* strain (Figure 2b,c; blue), suggesting that deletion of SrtA does not broadly activate compensatory expression pathways under these conditions. In contrast, numerous proteins were consistently enriched in the WT strain (Figure 2b,c; red), indicating their depletion in the Δ*srtA* mutant. Among these were multiple canonical SrtA substrates containing LPxTG sorting motifs. Importantly, the principal significantly altered proteins identified in these analyses remained significant following FDR correction (Supplementary Data S3 and S4). The high reproducibility of the two independent biological replicates is further illustrated by the identification of 1253 proteins common to both datasets, with only 26 and 65 proteins unique to biological replicates 1 and 2, respectively (Figure S1).

Among these, proteins containing experimentally validated or predicted LPxTG variants were consistently depleted in both datasets. These include SPD_0080, containing an LPNTG motif and predicted to function as an adhesin, and ZmpB, containing an LPQTG motif and functioning as a metalloprotease virulence factor [18,19,20]. Both proteins were identified with high confidence across biological replicates. Additional predicted SrtA-dependent surface proteins were also reduced in at least one dataset, including StrH (LPQTG), BgaA (LPNTG), IgA1 protease (LPNTG), and NanA (LPETG), each containing LPxTG-like motifs consistent with SrtA-mediated anchoring.

Interestingly, a subset of proteins lacking canonical LPxTG sorting motifs was also consistently depleted in the Δ*srtA* strain. These included the adjacent gene products SPD_0093, SPD_0094, SPD_0095, and SPD_0096, which comprise the previously characterized *ptv* (*phenotypic tolerance to vancomycin*) operon and were designated *ptvC*, *ptvB*, *ptvA*, and *ptvR*, respectively [21]. This locus is induced by vancomycin and has been implicated in phenotypic tolerance to cell-wall-targeting stress, with several of its encoded proteins predicted to be membrane associated. Also depleted was the fucose isomerase FucA, a component of the pneumococcal fucose-utilization pathway. Interestingly, the physiological role of this pathway remains incompletely understood, as *S. pneumoniae* encodes functional fucose-processing enzymes yet does not efficiently utilize fucose as a sole carbon source [22]. Because SPD_0093-SPD_0096 and FucA lack recognizable SrtA sorting motifs, their reduced abundance is unlikely to reflect direct SrtA-mediated anchoring but instead result from regulatory responses associated with disruption of the pneumococcal cell surface.

Together, these data demonstrate that deletion of SrtA produces the expected selective loss of LPxTG-bearing surface proteins despite only a modest reduction in bacterial growth. The observed proteomic changes therefore primarily reflect disruption of SrtA-dependent surface anchoring rather than a generalized consequence of impaired growth.

### 3.2. Production and Biochemical Characterization of Monomeric S. pneumoniae SrtA

**Figure 3:**
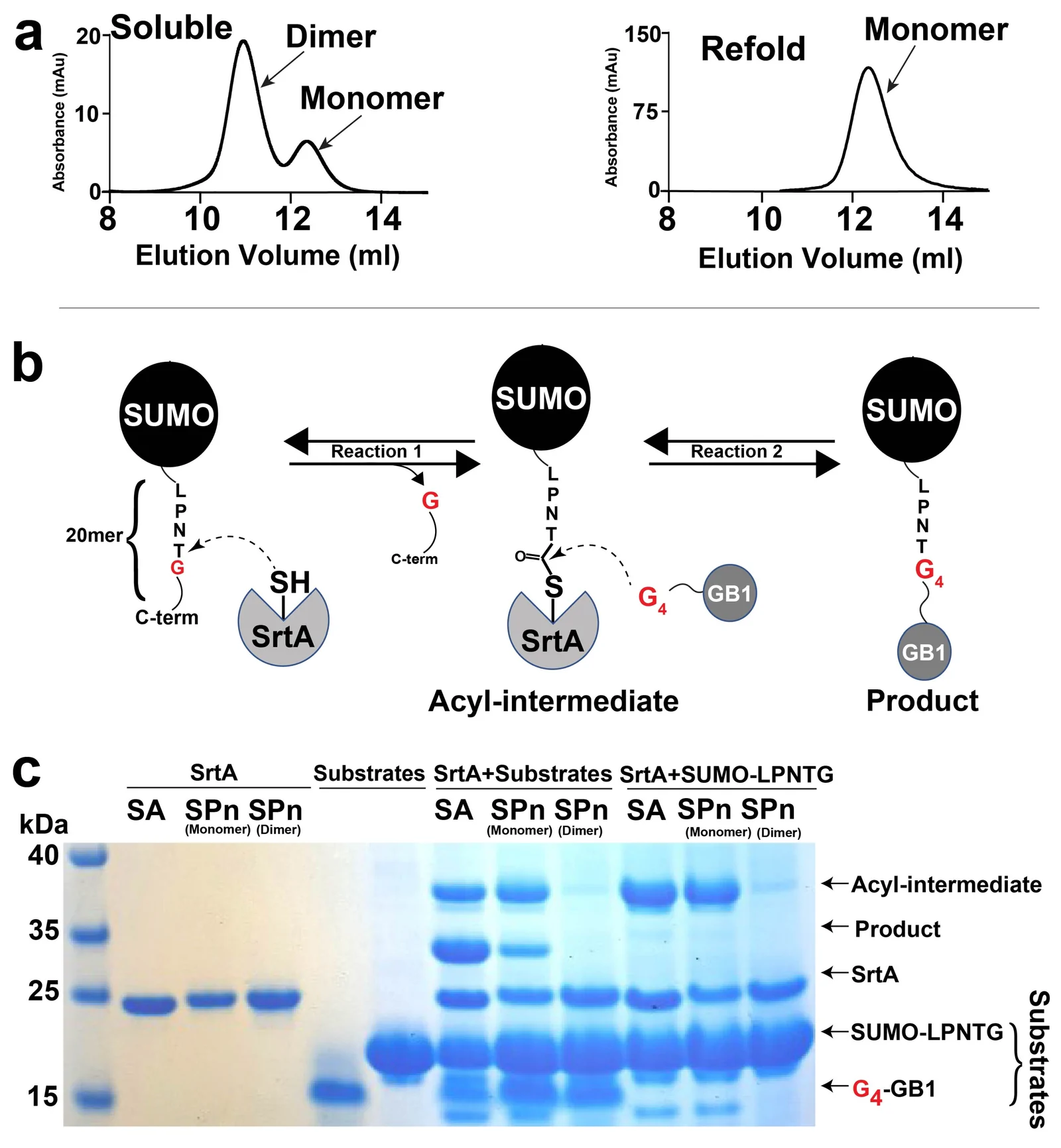
Purification and activity of *S. pneumoniae* SrtA. (**a**) Size-exclusion-chromatograms of purified soluble SPn SrtA (**left**) and the refolded SPn SrtA monomer (right). Elution profiles are shown from an analytical Superdex-75 (23.5 mL). (**b**) Schematic of our recombinant system to mimic the SrtA reactions in vitro (**top**) and the SDS-PAGE gel monitoring activity of *S. aureus* SrtA and both the monomer and dimer of *S. pneumoniae* SrtA with substrates and products delineated. (**c**) Gel-based catalytic assay of *S. aureus* SrtA (SA) and both the purified *S. pneumoniae* SrtA monomer and dimer (SPn). Catalysis was tested in the presence of both substrates, SUMO-LPNTG and G_4_-GB1, as well as only with the SUMO-LPNTG substrate. Corresponding molecular weights of markers are shown. SrtA concentrations were 250 μM with both substrate concentrations of 500 μM incubated at room temperature overnight.

To directly compare the catalytic properties of monomeric and domain-swapped S. pneumoniae SrtA, we expressed the soluble catalytic domain (residues 82–247), omitting the N-terminal membrane anchor (Figure S2). Consistent with the previously reported crystal structure [10], purification under native conditions predominantly yielded the domain-swapped dimer (Figure 3a, left). In contrast, purification under denaturing conditions followed by refolding using a guanidine/arginine dialysis strategy previously established in our laboratory yielded predominantly monomeric SrtA (Figure 3a, right). This approach differs from the previously reported “fast-refolding” procedure for *S. pneumoniae* SrtA. Specifically, this prior refolding procedure involved Ni-affinity purification of a denatured SrtA into a 1:100 diluted volume of refolding buffer and subsequent re-application to Ni-affinity column [11]. In contrast, here refolding is accomplished directly by dialysis after purifying the protein via Ni-affinity under denaturing conditions (see Materials and Methods). Our refolding strategy has proven broadly applicable to diverse proteins, including receptor domains and enzymes [15,16,17]. For *S. pneumoniae* SrtA here, independent refolding preparations produced highly similar SEC profiles dominated by the monomeric species, with only a minor earlier-eluting dimeric component (Figure S3). These chromatograms were obtained by direct injection of the refolded material before preparative SEC, demonstrating the reproducibility of the guanidine/arginine dialysis strategy. Together, the native purification and refolding protocols yielded preparations enriched in the domain-swapped dimer and monomer, respectively, enabling direct biochemical comparison of these two oligomeric states.

To compare the catalytic activities of monomeric and domain-swapped SrtA, we established a recombinant transpeptidation assay that recapitulates the two-step sortase reaction (Figure 3b). The assay utilizes two recombinant model substrates, one containing the LPNTG recognition motif and a second containing an N-terminal oligoglycine nucleophile. For the first substrate, we used a 6× His-tagged Small Ubiquitin-Like Modifier (SUMO) fused to a 20-residue peptide containing an LPNTG motif (SUMO-LPNTG). SrtA is expected to cleave this peptide between the threonine and glycine residues to generate a thioacyl-enzyme intermediate. As the second substrate, we used the B1 domain of Streptococcal Protein G (GB1) engineered with an N-terminal tetra-glycine sequence followed by a 20-residue linker (G_4_-GB1). The N-terminal oligoglycine serves as a model nucleophile, providing a free amino group analogous to that provided by the endogenous peptidoglycan precursor, Lipid II, which serves to resolve the thioacyl-enzyme intermediate during transpeptidation. Although the pneumococcal Lipid II contains a strain-dependent branched peptide initiated by serine or alanine rather than the pentaglycine found in *S. aureus*, G_4_-GB1 was employed as a generic oligoglycine nucleophile to model the transpeptidation reaction and facilitate biochemical characterization [23].

We utilized this gel-based assay to probe the catalytic activity of monomeric *S. pneumoniae* SrtA (Figure 3c). To this end, the soluble domain of the previously characterized *S. aureus* SrtA homologue (residues 60–206) was used as a positive control, and the *S. pneumoniae* SrtA dimer was included for comparison. The *S. pneumoniae* SrtA dimer and monomer were separately purified from a soluble purification and refolding purification, respectively. This assay demonstrates that both the *S. aureus* SrtA and the *S. pneumoniae* SrtA monomer produce both the acyl-intermediate and the covalently coupled product that ligates the SUMO-LPNTG with the G_4_-GB1. In contrast, no detectable activity was observed for the *S. pneumoniae* SrtA dimer under these assay conditions. Also as expected, when only the SUMO-LPNTG substrate is present, meaning without the G_4_-GB1, the acyl-intermediate alone is observed. The use of separately purified dimeric and monomeric preparations enabled direct assessment of the catalytic competence of each oligomeric state.

To further validate the identity of this acyl-intermediate species, the corresponding gel band was excised and analyzed by mass spectrometry (Supplementary Data S5). Although the thioacyl linkage itself could not be directly observed, peptide fragments corresponding to both the SUMO-20mer and *S. pneumoniae* SrtA were identified from this band, with 532 and 845 spectral counts, respectively. The simultaneous detection of SrtA and substrate within this gel-purified band supports the formation of a covalently linked sortase–substrate intermediate.

Finally, we also subjected the active *S. pneumoniae* SrtA to the standard FRET-based catalytic assay using the commercial Abz-LPETG-K(Dnp)-NH_2_ substrate (Figure S4). Whereas this assay employs the widely used LPETG recognition motif to facilitate comparison with previous sortase studies, our recombinant transpeptidation assay and NMR titrations directly utilize the LPNTG sequence found in several *S. pneumoniae* surface proteins. Similar catalytic parameters were obtained to those of *S. aureus* SrtA [24], consistent with the relatively poor activity of sortases in vitro [24]. Such low in vitro activity has been ascribed to the fact that in vivo catalysis occurs on a two-dimensional surface that brings substrates into close proximity with membrane-anchored sortases [6]. Thus, our guanidine/arginine refolding strategy provides a simple and reproducible means of obtaining catalytically competent monomeric *S. pneumoniae* SrtA for downstream biochemical and NMR studies.

### 3.3. The S. pneumoniae SrtA Predicted Structural Model Is Consistent with Its NMR Solution Resonance Assignments

Resonance assignments were obtained for the *S. pneumoniae* SrtA monomer (deposited in the BMRB as accession number 53397) and were used here to assess the quality of the AlphaFold predicted model. We anticipated a highly accurate prediction considering the overall ~30% sequence identity to other SrtA enzymes already determined and the fact that the swapped dimer structure was elucidated by X-ray crystallography [10]. Specifically, Cα resonances are particularly sensitive measures of the secondary structure, whereby their differences to that within a random coil for each amino acid type can be used to quantify whether residues have α-helical or β-strand propensities in solution. Cα chemical shift differences relative to a random coil (ΔCα) were plotted using random coil Cα values [25]. Positive differences indicate α-helical propensities while negative values indicate β-strand propensities that are both observed (Figure S5a; bottom). Residues exhibiting three or more values with similar Cα propensities were plotted onto the AlphaFold model, indicating that the solution secondary structure assessed by Cα resonances is largely in agreement (Figure S5a; top). The only disagreement between the measured secondary structure propensities and the predicted model was that of residues 189–193 that comprise a predicted helix known to fold over the active site within the β6/β7 loop. Specifically, while the structural model depicts a small helix, the Cα resonances exhibit little to slightly negative values that suggest this region largely samples a random coil in solution. This predicted β6/β7 helix is found in the X-ray crystal structure of the *S. pneumoniae* SrtA swapped dimer [10], within several *S. aureus* SrtA structures [26], and within other sortases as well [27,28]. However, the β6/β7 region is also non-helical in the apo solution NMR ensemble of *S. aureus* SrtA (PDB 1IJA), the first structure reported for a sortase enzyme [29]. Thus, the β6/β7 region can adopt multiple conformations, and our solution NMR data indicate that it predominantly samples a more extended, non-helical conformation in *S. pneumoniae* SrtA.

To further characterize this region, longitudinal (R1) and transverse (R2) relaxation measurements were obtained (Figure S5b,c). Residues within the β6/β7 loop, particularly multiple residues within 188–193, exhibit elevated R1 and R2 values relative to the protein average, consistent with increased dynamics within both the fast timescale (nanosecond) and slow timescales (micro-millisecond) of active-site loop, respectively. Together with the secondary chemical shift analysis, these data support increased conformational flexibility in the β6/β7 region relative to the AlphaFold prediction.

### 3.4. S. pneumoniae SrtA Binds Divalent Cations Despite Not Requiring Them for Catalytic Activity

**Figure 4:**
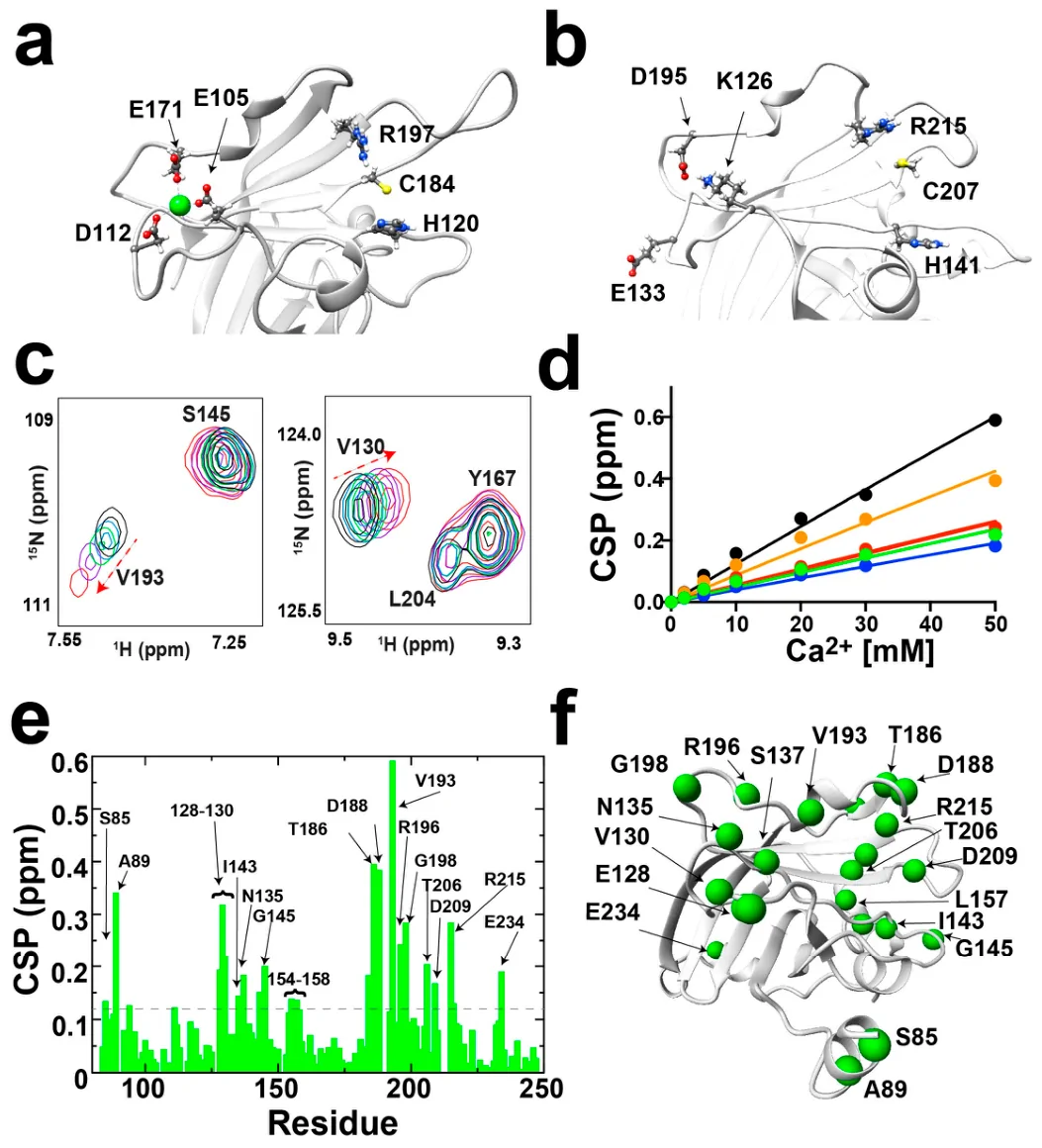
Monitoring the effects of Ca^2+^ with *S. pneumoniae* SrtA. (**a**) *S. aureus* SrtA active site indicating the Ca^2+^ (green sphere) coordinating residues E105, D112, and E171, along with the catalytic triad residues of H120, C184, and R197. (**b**) *S. pneumoniae* SrtA active site indicating the homologous residues to that of *S. aureus* SrtA, including residues K126, E133, D195 and the catalytic triad of H141, C207, and R215. (**c**) Selected regions of the *S. pneumoniae* SrtA ^15^N-HSQC titration with Ca^2+^. Spectra shown include 0 (black), 5 mM (sky blue), 10mM (green), 30 mM (mauve), and 50 mM (red) CaCl_2_ with resonance shifts highlighted (dashed red arrow). (**d**) Individual CSPs tracked over the indicated range of added Ca^2+^. Resonances include V130 (green), S137 (blue), T186 (orange), V193 (black), and R196 (red). (**e**) CSPs between *S. pneumoniae* SrtA in the absence and presence of 50 mM Ca^2+^. The average (0.083 ppm) plus half standard deviation (0.045) is delineated (dashed line). (**f**) CSPs induced by 50 mM Ca^2+^ higher than the average plus half the standard deviation (0.13 ppm) are mapped onto the SrtA model (green spheres).

Previous biochemical studies demonstrated that *S. pneumoniae* SrtA is Ca^2+^ independent [10,11]. We therefore sought to determine whether this lack of dependence reflects an inability to bind Ca^2+^ or, alternatively, weak nonessential metal binding. To address this question, we monitored Ca^2+^ binding directly by NMR titration. In *S. aureus* SrtA, Ca^2+^ is chelated by three sidechains that include E105, D112, and E171 (Figure 4a). In contrast, *S. pneumoniae* SrtA comprises D195, E133, and K126 (Figure 4b). Structurally, *S. pneumoniae* SrtA is most similar to *S. pyogenes* SrtA that also does not engage Ca^2+^, which is likely due to similar residues at the potential of Ca^2+^ binding site. In fact, although *S. pyogenes* SrtA does not need Ca^2+^ for catalysis, this metal does slow the kinetics of catalysis, suggesting that weak Ca^2+^ binding may still persist but serves to block a competent formation of the active site [27].

**Figure 5:**
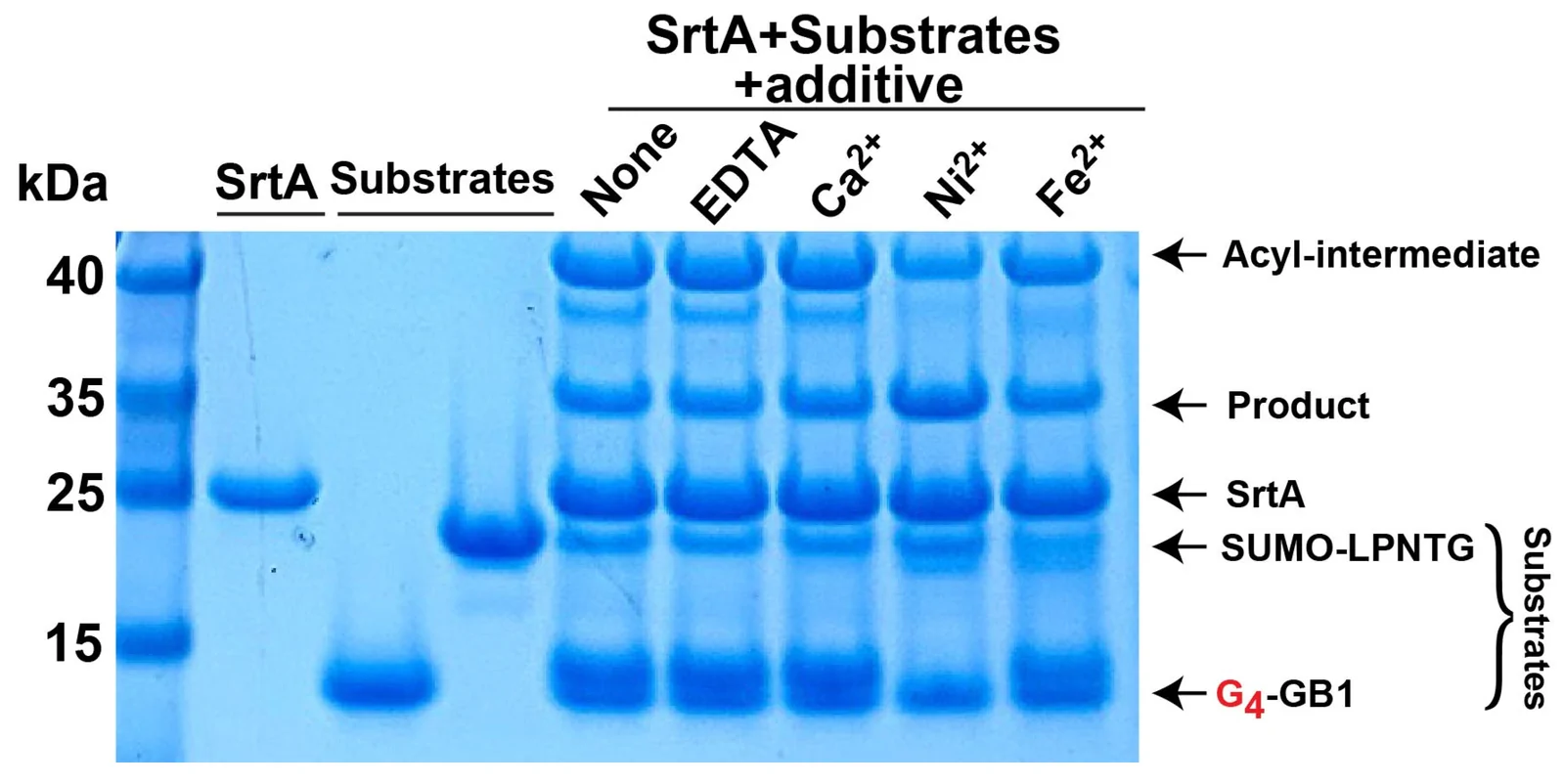
*S. pneumoniae* SrtA catalytic activity is metal independent. Gel-based activity assay in the absence and presence of metals. From left to right, *S. pneumoniae* SrtA is shown alone, both substrates alone that include G_4_-GB1 and SUMO-LPNTG, SrtA and both substrates with no metal added (None), 5 mM EDTA added, 5 mM Ca^2+^, 5 mM Ni^2+^, and 5 mM Fe^2+^.

We directly tested *S. pneumoniae* SrtA binding to Ca^2+^ using NMR titrations. Despite previous studies that have shown Ca^2+^ independence for *S. pneumoniae* SrtA [11], Ca^2+^ binding was found to perturb selective resonances (Figure 4c and Figure S6). However, the affinity to Ca^2+^ was not saturable, as shown by generating binding isotherms as a function of Ca^2+^ up to 50 mM of the metal (Figure 4d), consistent with a very weak affinity. Most residues affected by Ca^2+^ are relegated to the *S. pneumoniae* SrtA active site (Figure 4e,f). Such a weak affinity of *S. pneumoniae* SrtA to Ca^2+^ is consistent with prior studies that show this sortase is not dependent on such a metal for catalysis. As expected, our gel-based catalytic assay clearly illustrated that the transpeptidase reaction of SUMO-LPNTG to G_4_-GB1 was not altered by Ca^2+^ or other metals (Figure 5. In fact, this independence from metals was further corroborated by observed activity in the presence of EDTA (Figure 5). Together, these results explain the previously observed calcium independence of *S. pneumoniae* SrtA by demonstrating that Ca^2+^ binds only weakly and is not required for catalytic activity.

During these studies, purified *S. pneumoniae* SrtA was found to engage Ni^2+^ during its purification (Figure S7), suggesting a relatively high affinity to this metal during the Ni-affinity purification. This was first noted by the identification of two resonances for over a dozen residues (Figure S7a,b), which resulted in duplicate resonances during assignments (Figure S7c). Considering that the Cα resonances were identical and that Cα resonances are sensitive to secondary structure, this suggested that only the local chemical environments differed between a free and bound form. Subsequent purification with EDTA led to a single set of resonances and the reintroduction of Ni^2+^ shifted these resonances towards the secondary resonances initially observed (Figure S7d). Although the physiological significance of this interaction remains unknown, metal homeostasis is increasingly recognized as a central determinant of *S. pneumoniae* physiology and virulence, particularly for transition metals such as Mn, Zn, Fe, and Cu [30]. Whether Ni^2+^ similarly contributes to these processes or instead represents an adventitious interaction during recombinant purification remains to be determined. Importantly, catalytic assays revealed no dependence on Ni^2+^ for transpeptidase activity, indicating that this interaction does not directly contribute to catalysis under the conditions examined.

### 3.5. S. pneumoniae SrtA Engages the Canonical Recognition Motif

To directly assess recognition of the canonical LPxTG motif by *S. pneumoniae* SrtA, we performed NMR titrations using a 9-mer peptide (SYELPNTGS). This explicit peptide sequence encompasses the LPNTG sequence found in several *S. pneumoniae* surface proteins, including IgA1 protease (IgA1P) and β-galactosidase A (BgaA), both identified in our proteomic analyses above.

**Figure 6:**
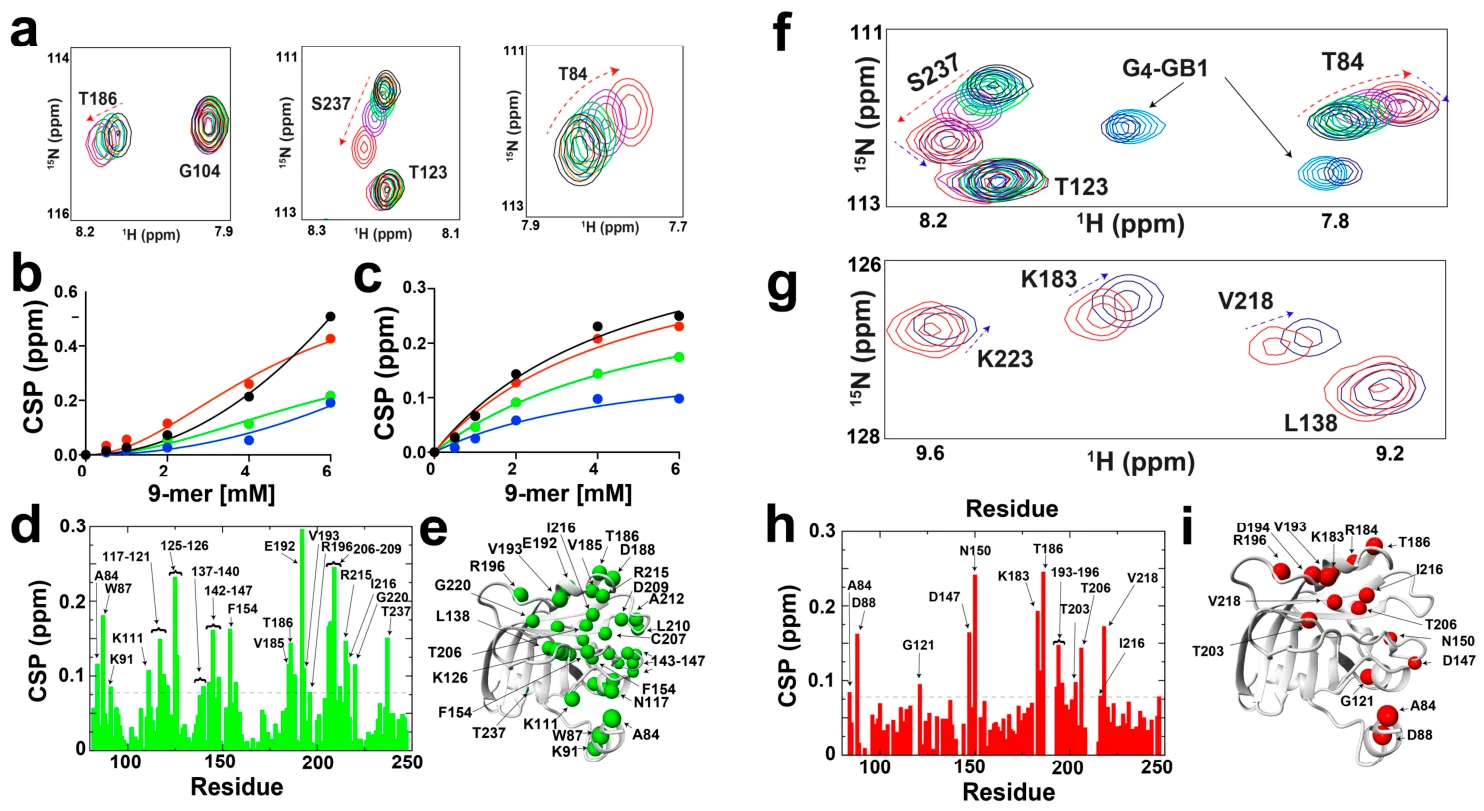
*S. pneumoniae* SrtA interactions with substrate. (**a**) Selected regions of the *S. pneumoniae* SrtA ^15^N-HSQC titration with a substrate 9-mer indicating linear resonance shifts that increase with substrate (**left**), linear resonance shifts that are saturable (**middle**), and curved shifts (**right**). Substrate concentrations were 0 mM (black), 1 mM (orange), 2 mM (green), 4 mM (mauve), and 6 mM (red). (**b**) Extracted binding isotherms for amide resonance shifts that were unsaturable, which include S229 (black), T84 (red), Y247 (blue), and L107 (green). Shifts were globally fit using a random model with a Hill coefficient simply for visualization. (**c**) Extracted binding isotherms for amide resonance shifts that were saturable, which include T186 (black), K126 (red), G112 (blue), and I157 (green). Shifts were globally fit with an estimated K_D_ of 5 ± 1 mM. (**d**) CSPs between *S. pneumoniae* SrtA in the absence and presence of 2 mM 9-mer. The average (0.052 ppm) plus half standard deviation (0.026) is delineated (dashed line). (**e**) CSPs induced by 2 mM 9-mer higher than the average plus half the standard deviation (0.078 ppm) are mapped onto the SrtA model (green spheres). (**f**) Selected region of the *S. pneumoniae* SrtA ^15^N-HSQC titration with a substrate 9-mer with CSPs delineated (dashed red arrow) and in the presence of 6 mM G_4_-GB1 with CSPs also delineated (dashed navy arrow). Shown is the substrate 9-mer titration in the same colors as above with the addition of 6 mM unlabeled G_4_-GB1 (navy blue) and ^15^N-labeled G_4_-GB1 (sky blue). Resonances emanating from the G_4_-GB1 are delineated. (**g**) Selected region of the *S. pneumoniae* SrtA ^15^N-HSQC titration showing only resonances with either 6 mM of the substrate 9-mer alone (red) or both substrates that include the 6 mM G_4_-GB1 (navy blue). Arrows delineate shift directions (dashed blue lines). (**h**) CSPs between *S. pneumoniae* SrtA with 6 mM 9-mer in the absence and presence of 6 mM G_4_-GB1. The average (0.057 ppm) plus half standard deviation (0.021 ppm) is delineated (dashed line). (**i**) CSPs induced by G_4_-GB1 higher than the average plus half the standard deviation (0.078 ppm) are mapped onto the SrtA model (red spheres).

NMR titrations revealed a heterogeneous mixture of chemical shift perturbation (CSP) patterns, including both saturable and non-saturable responses (Figure 6a). The non-linear behavior observed for many residues suggests that peptide binding is accompanied by conformational redistribution within the enzyme, preventing the full dataset from being adequately described by a simple two-state binding model (Figure 6b). Nevertheless, a subset of resonances displayed clear, saturable binding isotherms (Figure 6c), allowing global fitting and yielding an apparent dissociation constant (K_D_) of approximately 5 mM. This affinity falls within the expected range for sortase-substrate interactions, which are typically weak. For example, *S. aureus* SrtA binds its LPxTG recognition motif with even lower affinity [24].

Notably, many resonances displayed pronounced line broadening upon substrate addition, likely reflecting accumulation of the covalent thioacyl intermediate at higher peptide concentrations. This increase in intermediate formation may also contribute to the heterogeneous CSP profiles and possibly substrate-induced unfolding. To provide a consistent view, CSPs resulting from 2 mM peptide are shown (Figure 6d,e). These shifts predominantly localized to the active site and surrounding regions, consistent with engagement of the LPNTG motif by the β6/β7 region.

We further examined the ability of SrtA to accommodate a second substrate by titrating G_4_-GB1 in the presence of excess 9-mer peptide. Specifically, 6 mM G_4_-GB1 was added in the presence of 6 mM LPNTG peptide (Figure 6f). Additional resonances appeared in the HSQC spectrum; ^15^N-labeled G_4_-GB1 control spectra confirmed these peaks were from the unbound substrate. Nevertheless, numerous SrtA resonances showed distinct CSPs upon G_4_-GB1 addition (Figure 6g). Despite extensive line broadening induced by the 9-mer, CSPs caused by G_4_-GB1 addition again mapped to the same active site region (Figure 6h,i), further supporting productive engagement at this site.

## 4. Discussion

### 4.1. SrtA as a Virulence Factor in S. pneumoniae

SrtA plays a central role in *S. pneumoniae* pathogenesis by anchoring LPxTG-containing surface proteins that mediate adhesion, colonization, and immune evasion. These substrates include NanA, BgaA, ZmpB metalloprotease, and the IgA1 protease, each of which contributes to host interaction and virulence. Deletion of *srtA* was already shown to significantly impair pneumococcal colonization and reduce virulence in murine infection models, demonstrating its critical role in both nasopharyngeal persistence and invasive disease [9]. Furthermore, immunization with recombinant *S. pneumoniae* SrtA protects mice against lethal challenge, highlighting its promise as a vaccine antigen [31].

Consistent with these prior observations, our proteomic profiling reinforces the essential role of SrtA in maintaining the pneumococcal surface proteome. Quantitative mass spectrometry comparing WT and Δ*srtA* strains revealed that multiple LPxTG-containing proteins were absent in the knockout background. These include canonical adhesins and virulence factors such as IgA1 protease, ZmpB, PavB, and NanA, as well as several uncharacterized LPxTG-bearing proteins predicted to be cell-wall-associated. Additionally, several non-LPxTG proteins were also reproducibly depleted, most notably SPD_0093–SPD_0096, which comprise the *ptv* operon implicated in phenotypic tolerance to cell-wall-targeting stress [21]. Their coordinated depletion reveals that loss of SrtA-dependent surface anchoring induces secondary consequences for cell-envelope homeostasis. FucA was also depleted, suggesting that these consequences may extend to cellular metabolism. However, while FucA is a component of the pneumococcal fucose-utilization pathway, the physiological role of this pathway and the specific role of FucA remains incompletely understood. This is because *S. pneumoniae* possesses functional fucose-processing machinery but does not efficiently utilize fucose as a sole carbon source, which has led to speculation of non-catalytic roles for FucA [22]. One possibility is that disruption of the SrtA-dependent cell surface is sensed through cell-envelope stress-responsive regulatory systems, thereby producing secondary changes in both envelope-associated and metabolic pathways. Together, these changes suggest broader physiological remodeling following loss of SrtA, although the mechanisms linking surface protein anchoring to these secondary effects remain to be determined.

Together, these findings reinforce the central role of SrtA in organizing the pneumococcal surface proteome. The loss of LPxTG-bearing surface proteins in the Δ*srtA* strain underscores its role in maintaining the adhesive and immunomodulatory capacity of the bacterial envelope. These biological observations, in combination with previous murine and cellular infection studies, establish SrtA as a central virulence determinant and a compelling target for both therapeutic and vaccine development.

### 4.2. Biochemical and Structural Characterization of Monomeric S. pneumoniae SrtA

While active monomeric *S. pneumoniae* SrtA has previously been obtained by refolding [11], the guanidine/arginine dialysis strategy described here provides a simple and reproducible approach that consistently generates preparations enriched in the catalytically active monomer. This protocol proved sufficiently robust for biochemical assays and high-resolution NMR spectroscopy, enabling direct comparison of the active monomer with the inactive domain-swapped dimer. Using these preparations, we developed a recombinant transpeptidation assay that directly demonstrates cleavage and transpeptidation by monomeric SrtA. The assay employs a SUMO–LPNTG fusion protein containing the canonical LPxTG recognition motif together with a G_4_-GB1 nucleophile that models the nucleophilic component of the transpeptidation reaction. In addition to validating monomeric SrtA activity, this assay revealed no detectable activity of the isolated domain-swapped dimer. Together with the NMR titrations, these experiments establish productive recognition of canonical LPxTG substrates and provide the first residue-level description of substrate engagement by active *S. pneumoniae* SrtA. The heterogeneous CSP profiles may further suggest that peptide recognition is coupled to conformational redistribution within the enzyme rather than a simple two-state binding event.

In agreement with previous biochemical studies [11], catalysis was independent of Ca^2+^ despite weak detectable interactions observed by NMR. The present work further identified a previously unrecognized reversible interaction with Ni^2+^. Unlike Ca^2+^, Ni^2+^ remained associated with SrtA throughout purification and was only removed by EDTA, indicating tight retention by the protein. Although the physiological significance of this interaction remains unknown, the increasing appreciation of transition-metal homeostasis in *S. pneumoniae* suggests that this observation may warrant further investigation. Importantly, Ni^2+^ was not required for transpeptidase activity, indicating that metal binding and catalysis are uncoupled under the conditions examined.

Beyond establishing a practical platform for biochemical and NMR studies, the present system provides opportunities for future investigations of substrate recognition, conformational dynamics, covalent reaction intermediates, and inhibitor discovery directed toward this important pneumococcal virulence determinant.

### 4.3. S. pneumoniae SrtA Domain Swapping and the Active Monomer

The ability to reproducibly isolate catalytically active monomeric *S. pneumoniae* SrtA by refolding suggests that the previously reported domain-swapped dimer represents an alternative folding state [10], rather than the physiologically relevant or catalytically competent conformation. The molecular basis for this three-dimensional domain swapping remains unclear but may reflect a competing folding pathway favored during recombinant expression, purification, or crystallization. Although the biological significance of this alternative conformation remains unknown, its reproducible formation and subsequent conversion to an active monomer provide a unique opportunity to investigate the structural determinants governing domain swapping. Given that hundreds of proteins are now known to undergo three-dimensional domain swapping [32], *S. pneumoniae* SrtA represents a useful experimental system for elucidating the folding pathways and structural features that promote this widespread structural phenomenon.

## 5. Conclusions

In this study, we establish a biochemical and structural framework for investigating the active form of *S. pneumoniae* SrtA. Proteomic analysis of the ΔsrtA strain confirmed the loss of multiple LPxTG-containing surface proteins, reinforcing the central role of SrtA in pneumococcal surface protein anchoring. Using a simple and reproducible guanidine/arginine refolding strategy, we generated catalytically active monomeric SrtA that enabled direct comparison with the previously characterized inactive domain-swapped dimer. Biochemical assays confirmed LPxTG cleavage and transpeptidation by the monomer, while NMR spectroscopy provided the first residue-level characterization of substrate recognition and active-site dynamics in the active enzyme. Together, these findings provide a robust platform for future mechanistic, structural, and inhibitor studies of *S. pneumoniae* SrtA and further support this essential virulence factor as a promising therapeutic target.

## Data Availability

NMR backbone resonance assignments for *S. pneumoniae* SrtA have been deposited in the Biological Magnetic Resonance Data Bank (BMRB accession number 53397). Backbone resonance assignments were completed for >95% of residues in the 82–247 construct of *S. pneumoniae* SrtA, with >97% of HN, N, Cα, and Cβ resonances assigned. The mass spectrometry proteomics data supporting the comparisons of WT and Δ*srtA* strains have been deposited in the PRIDE repository under accession number PXD082607. All other data supporting the findings of this study are available within the article and its Supplementary Information or from the corresponding author upon reasonable request.

## Supplementary Materials

The following supporting information can be downloaded at: https://www.mdpi.com/article/10.3390/biom16091231/s1. Figure S1: Reproducibility of the quantitative proteomic analyses; Figure S2. Comparison between *S. pneumoniae* SrtA and S. aureus SrtA; Figure S3. Reproducibility of monomeric *S. pneumoniae* SrtA production by guani-dine/arginine refolding. Figure S4. *S. pneumoniae* SrtA catalysis of Abz-LPETG-K(Dnp)-NH_2_; Figure S5. Cα propensities largely agree with the predicted model of S. pneumoniae SrtA; Figure S6. NMR titration of *S. pneumoniae* SrtA with Ca^2+^; Figure S7. *S. pneumoniae* SrtA binds Ni; Figure S8. Full, uncropped SDS–PAGE gels corresponding to figures in the main text; Data S1: Mass spectrometry results and data used for the volcano plot data for ΔsrtA versus wild-type cells (Figure 2a); Data S2: Mass spectrometry results and data used for the volcano plot data for ΔsrtA versus wild-type cells (Figure 2b); Data S3: Mass spectrometry results using FDR-adjusted *p* values for Supplementary Data S1; Data S4: Mass spectrometry results using FDR-adjusted *p* values for Supplementary Data S2; Data S5: Peptide identifications from LC-MS/MS analysis of the excised acyl-intermediate band corresponding to active *S. pneumoniae* SrtA (Figure 3c).

## References

[1] Breijyeh Z., Jubeh B., Karaman R. (2020). Resistance of Gram-Negative Bacteria to Current Antibacterial Agents and Approaches to Resolve It. Molecules.

[2] Ramirez J.A., Wiemken T.L., Peyrani P., Arnold F.W., Kelley R., Mattingly W.A., Nakamatsu R., Pena S., Guinn B.E., Furmanek S.P. (2017). Adults Hospitalized With Pneumonia in the United States: Incidence, Epidemiology, and Mortality. Clin. Infect. Dis..

[3] Kim G.L., Seon S.H., Rhee D.K. (2017). Pneumonia and *Streptococcus pneumoniae* vaccine. Arch. Pharm. Res..

[4] Gamez G., Castro A., Gomez-Mejia A., Gallego M., Bedoya A., Camargo M., Hammerschmidt S. (2018). The variome of pneumococcal virulence factors and regulators. Bmc Genom..

[5] Pallen M.J., Lam A.C., Antonio M., Dunbar K. (2001). An embarrassment of sortases—A richness of substrates?. Trends Microbiol..

[6] Jacobitz A.W., Kattke M.D., Wereszczynski J., Clubb R.T., KarabenchevaChristova T. (2017). Sortase Transpeptidases: Structural Biology and Catalytic Mechanism. Structural and Mechanistic Enzymology.

[7] Marks L.R., Reddinger R.M., Hakansson A.P. (2014). Biofilm formation enhances fomite survival of *Streptococcus pneumoniae* and Streptococcus pyogenes. Infect. Immun..

[8] Chen S., Paterson G., Tong H., Mitchell T., Demaria T. (2005). Sortase A contributes to pneumococcal nasopharyngeal colonization in the chinchilla model. FEMS Microbiol. Lett..

[9] Paterson G., Mitchell T. (2006). The role of *Streptococcus pneumoniae* sortase A in colonisation and pathogenesis. Microbes Infect..

[10] Biswas T., Misra A., Das S., Yadav P., Ramakumar S., Roy R.P. (2020). Interrogation of 3D-swapped structure and functional attributes of quintessential Sortase A from *Streptococcus pneumoniae*. Biochem. J..

[11] Nikghalb K.D., Horvath N.M., Prelesnik J.L., Banks O.G.B., Filipov P.A., Row R.D., Roark T.J., Antos J.M. (2018). Expanding the Scope of Sortase-Mediated Ligations by Using Sortase Homologues. Chembiochem.

[12] Tian B.-X., Eriksson L.A. (2011). Catalytic Mechanism and Roles of Arg197 and Thr183 in the Staphylococcus aureus Sortase A Enzyme. J. Phys. Chem. B.

[13] Wen Z., Sertil O., Cheng Y., Zhang S., Liu X., Wang W.-C., Zhang J.-R. (2015). Sequence Elements Upstream of the Core Promoter Are Necessary for Full Transcription of the Capsule Gene Operon in *Streptococcus pneumoniae* Strain D39. Infect. Immun..

[14] Li Y., Thompson C.M., Lipsitch M. (2014). A modified Janus cassette (Sweet Janus) to improve allelic replacement efficiency by high-stringency negative selection in *Streptococcus pneumoniae*. PLoS ONE.

[15] Redzic J.S., Armstrong G.S., Isern N.G., Jones D.N.M., Kieft S.K., Eisenmesser E. (2011). The retinal specific EMMPRIN/CD147 domain: From molecular structure to biological activity. J. Mol. Biol..

[16] Paukovich N., Xue M.J., Elder J.R., Redzic J.S., Blue A., Pike H., Miller B.G., Pitts T.M., Pollock D.D., Hansen K. (2018). Biliverdin Reductase B Dynamics Are Coupled to Coenzyme Binding. J. Mol. Biol..

[17] Lee E., Tran N., Redzic J.S., Singh H., Alamillo L., Holyoak T., Hamelberg D., Eisenmesser E.Z. (2025). Identifying and controlling inactive and active conformations of a serine protease. Sci. Adv..

[18] Blue C.E., Paterson G.K., Kerr A.R., Berge M., Claverys J.P., Mitchell T.J. (2003). ZmpB, a novel virulence factor of *Streptococcus pneumoniae* that induces tumor necrosis factor alpha production in the respiratory tract. Infect. Immun..

[19] Hsieh Y.C., Tsao P.N., Chen C.L., Lin T.L., Lee W.S., Shao P.L., Lee C.Y., Hsueh P.R., Huang L.M., Wang J.T. (2008). Establishment of a young mouse model and identification of an allelic variation of zmpB in complicated pneumonia caused by *Streptococcus pneumoniae*. Crit. Care Med..

[20] Gong Y., Xu W., Cui Y., Zhang X., Yao R., Li D., Wang H., He Y., Cao J., Yin Y. (2011). Immunization with a ZmpB-based protein vaccine could protect against pneumococcal diseases in mice. Infect. Immun..

[21] Liu X., Li J.W., Feng Z., Luo Y., Veening J.W., Zhang J.R. (2017). Transcriptional Repressor PtvR Regulates Phenotypic Tolerance to Vancomycin in *Streptococcus pneumoniae*. J. Bacteriol..

[22] Higgins M.A., Suits M.D., Marsters C., Boraston A.B. (2014). Structural and Functional Analysis of Fucose-Processing Enzymes from *Streptococcus pneumoniae*. J. Mol. Biol..

[23] Vollmer W., Massidda O., Tomasz A. (2019). The Cell Wall of *Streptococcus pneumoniae*. Microbiol. Spectr..

[24] Frankel B.A., Kruger R.G., Robinson D.E., Kelleher N.L., McCafferty D.G. (2005). Staphylococcus aureus sortase transpeptidase SrtA: Insight into the kinetic mechanism and evidence for a reverse protonation catalytic mechanism. Biochemistry.

[25] Hafsa N.E., Wishart D.S. (2014). CSI 2.0: A significantly improved version of the Chemical Shift Index. J. Biomol. NMR.

[26] Suree N., Liew C.K., Villareal V.A., Thieu W., Fadeev E.A., Clemens J.J., Jung M.E., Clubb R.T. (2009). The structure of the Staphylococcus aureus sortase-substrate complex reveals how the universally conserved LPXTG sorting signal is recognized. J. Biol. Chem..

[27] Race P.R., Bentley M.L., Melvin J.A., Crow A., Hughes R.K., Smith W.D., Sessions R.B., Kehoe M.A., McCafferty D.G., Banfield M.J. (2009). Crystal structure of Streptococcus pyogenes sortase A: Implications for sortase mechanism. J. Biol. Chem..

[28] Tamai E., Sekiya H., Maki J., Nariya H., Yoshida H., Kamitori S. (2017). X-ray structure of Clostridium perfringens sortase B cysteine transpeptidase. Biochem. Biophys. Res. Commun..

[29] Ilangovan U., Ton-That H., Iwahara J., Schneewind O., Clubb R.T. (2001). Structure of sortase, the transpeptidase that anchors proteins to the cell wall of Staphylococcus aureus. Proc. Natl. Acad. Sci. USA.

[30] Sanchez-Rosario Y., Durckel M., Meas R., Rohilla M., Parate S., Senanayaka S., Cota Ibarra J.A., Wierzbicki I.H., Gonzalez D.J., Johnson M.D.L. (2026). Iron and its import systems enhance copper accumulation in *Streptococcus pneumoniae*. mSphere.

[31] Gianfaldoni C., Maccari S., Pancotto L., Rossi G., Hilleringmann M., Pansegrau W., Sinisi A., Moschioni M., Masignani V., Rappuoli R. (2009). Sortase A Confers Protection against *Streptococcus pneumoniae* in Mice. Infect. Immun..

[32] Shameer K., Shingate P.N., Manjunath S.C., Karthika M., Pugalenthi G., Sowdhamini R. (2011). 3DSwap: Curated knowledgebase of proteins involved in 3D domain swapping. Database.

