# Supplementary material for: Structural and Functional Interrogation of Active Streptococcus pneumoniae Sortase A
Source: Biomolecules. 2026 Aug 25;16(9):1231. doi: 10.3390/biom16091231 (PMC13604702; doi:10.3390/biom16091231)
Supplement: Supplementary file 1 [file biomolecules-16-01231-s001.zip › biomolecules-4485780-supplementary.pdf]

biomolecules

MDPI

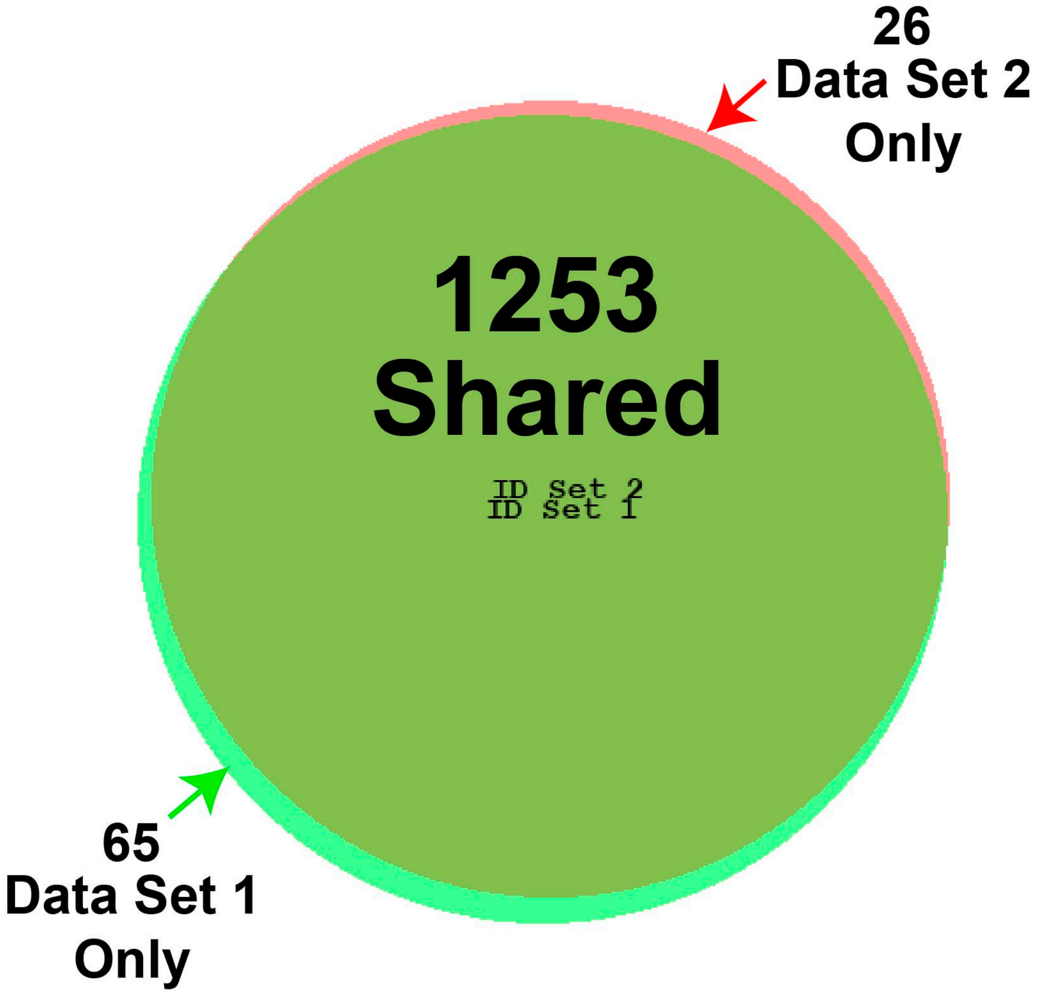

Figure S1. Reproducibility of the quantitative proteomic analyses. Venn diagram showing the overlap of proteins that passed quantitative filtering in the two independent biological proteomic datasets. A total of 1,279 and 1,318 proteins were quantified in biological datasets 1 (ID Set 1) and 2 (ID Set 2), respectively, with 1,253 proteins common to both datasets. Only 65 and 26 proteins were uniquely quantified in datasets 1 and 2, respectively, demonstrating the high reproducibility and extensive overlap of the two independent analyses.

a

```
S.pneumo:MSQKNNKKKNKRKNLLTNILAGFLILLSLALIFNTQIRNIFIVWNTNKYQVSQVSKEKLE 60
S.aureus:--------MKKWTNRLM-TIAGVVLILVAAYLFAKPHIDNYL--------HDKDKDEKIE 43
                  :* .* *   :**.:::*  * :* .   : ::         .: ..**:*

S.pneumo:ENQDTEGNFDFDSVKAISSEAVLTSQWDAQKLPVIGGIAIPELEMNLPIFKGLDNVNLFY 120
S.aureus:QYD---KNVKEQ--ASKDKKQQAKPQIPKDKSKVAGYIEIPDADIKEPVYPGPATPEQLN 98
         : :    *.. :   : ..:   . *   :*  * * * **: ::: *:: *  . : :

S.pneumo:-GAGTMKREQVMGEGNYSLASHHIFGVDNANKMLFSPLDNAKNGMKIYLTDKNKVYTYEI 179
S.aureus:RGVSFAEENESLDDQNISIAGHTFID---RPNYQFTNLKAAKKGSMVYFKVGNETRKYKM 155
          *..  :.:: :.: * *:*.* ::.     :  *: *. **:*  :*:.  *:. .*::

S.pneumo:REVKRVTPDRVDEVDDRDGV-NEITLVTCEDLAATERIIVKGDLKETKDYSQTSDEILTA 238
S.aureus:TSIRDVKPTDVGVLDEQKGKDKQLTLITCDDYNEKTGVWEKRKIFVATEVK--------- 206
          .:: *.*  *. :*::.*  :::**:**:*   .  :  * .:  :.: .

S.pneumo:FNQPYKQFY  247
S.aureus:---------  206
```

b

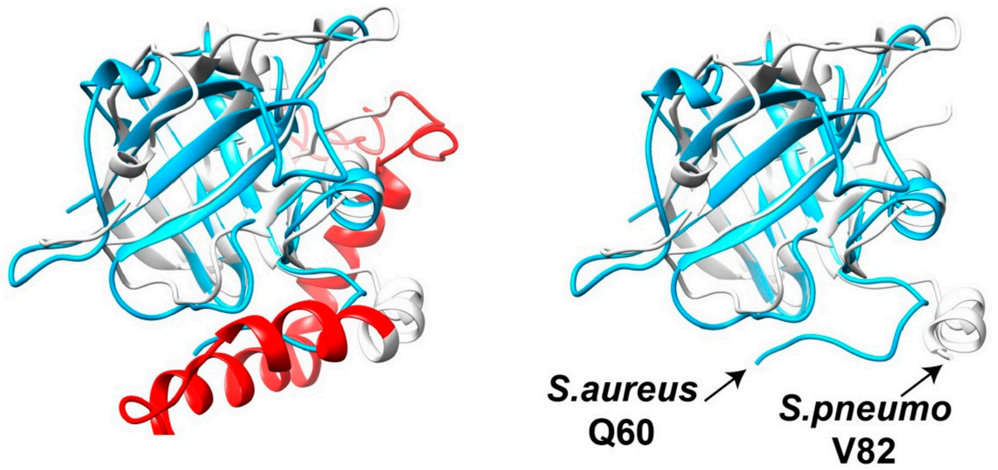

Figure S2. Comparison between *S. pneumoniae* SrtA and *S. aureus* SrtA. (**a**) Sequence alignment of full-length *S. pneumoniae* SrtA and *S. aureus* SrtA. The N-terminal regions that link the folded SrtA to the bacterial membrane are also delineated (red). (**b**) Structural alignment of the AlphaFold predicted *S. pneumoniae* SrtA (white) and the X-ray crystal structure of *S. aureus* SrtA (cyan, PDB: 1T2P). Left: The full *S. pneumoniae* SrtA predicted structure is shown with the N-terminal region (red). Right: Only *S. pneumoniae* SrtA residues 82-247 are shown.

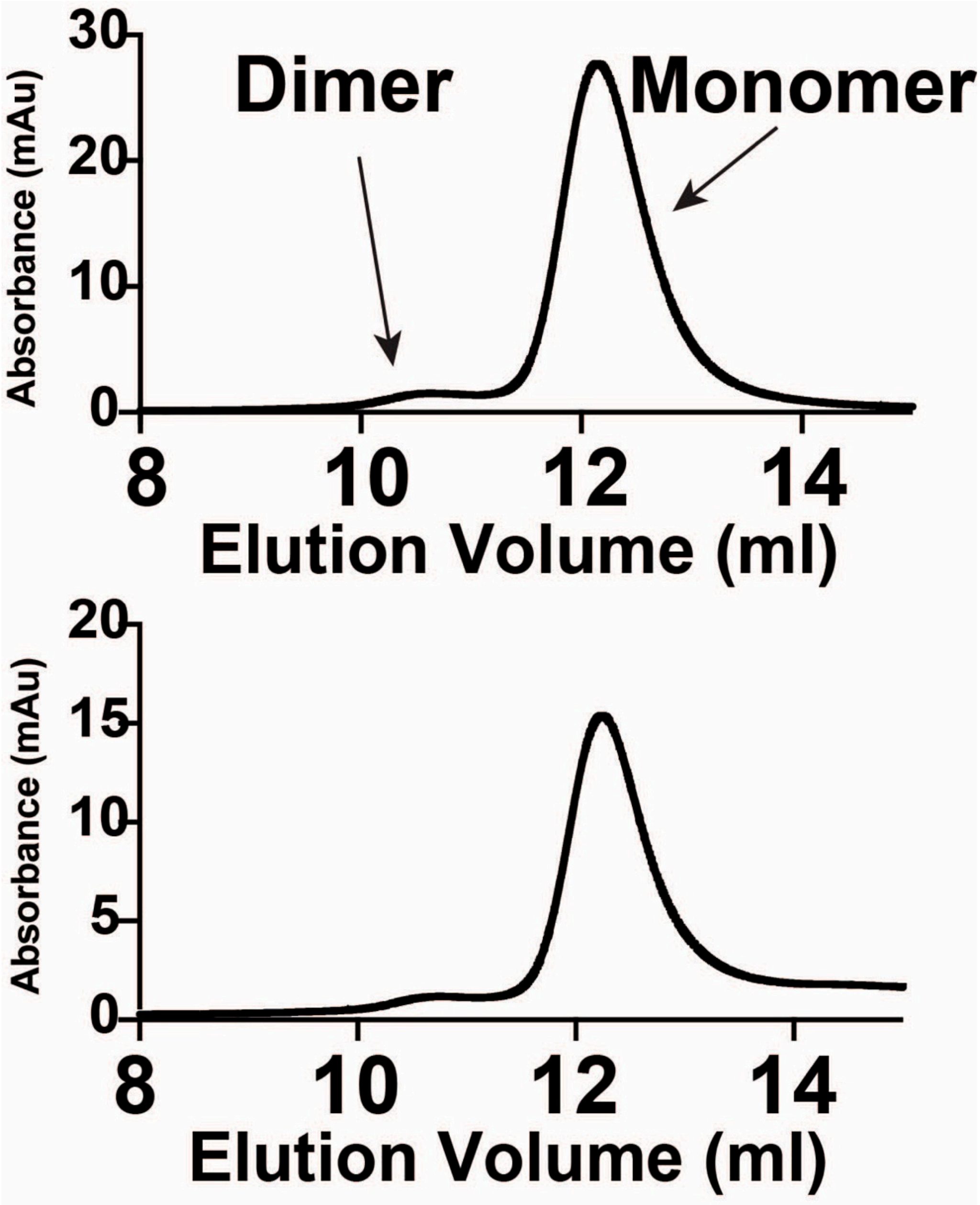

Figure S3. Reproducibility of monomeric *S. pneumoniae* SrtA production by guanidine/arginine refolding. Representative analytical SEC profiles from two independent refolding preparations are shown. Samples were injected directly following dialysis from denaturing conditions into final buffer, prior to preparative SEC purification. In both preparations, the predominant peak eluted at the volume expected for monomeric SrtA, with only a minor earlier-eluting species corresponding to the dimer.

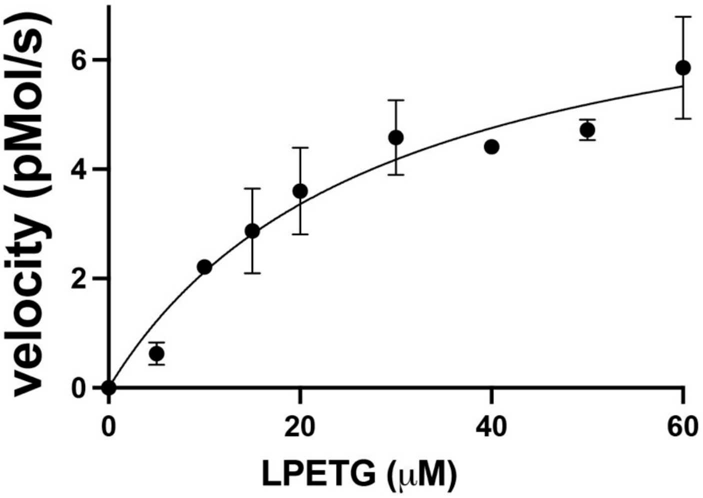

Figure S4. S. pneumoniae SrtA catalysis of Abz-LPETG-K(Dnp)-NH_2. *S. pneumoniae* SrtA was used at 10 μM with the indicated substrate concentrations in a final reaction volume of 200 μL. Data were fit to standard Michaelis–Menten kinetics using GraphPad Prism. Extracted parameters were a Vmax of 8.1 ± 1.0 pmol s^{-1} and a K_M of 28.4 ± 7.9 μM, corresponding to a k_{cat} of 0.0041 ± 0.0005 s^{-1} and a catalytic efficiency (k_{cat}/K_M) of approximately 0.14 mM^{-1} s^{-1}.

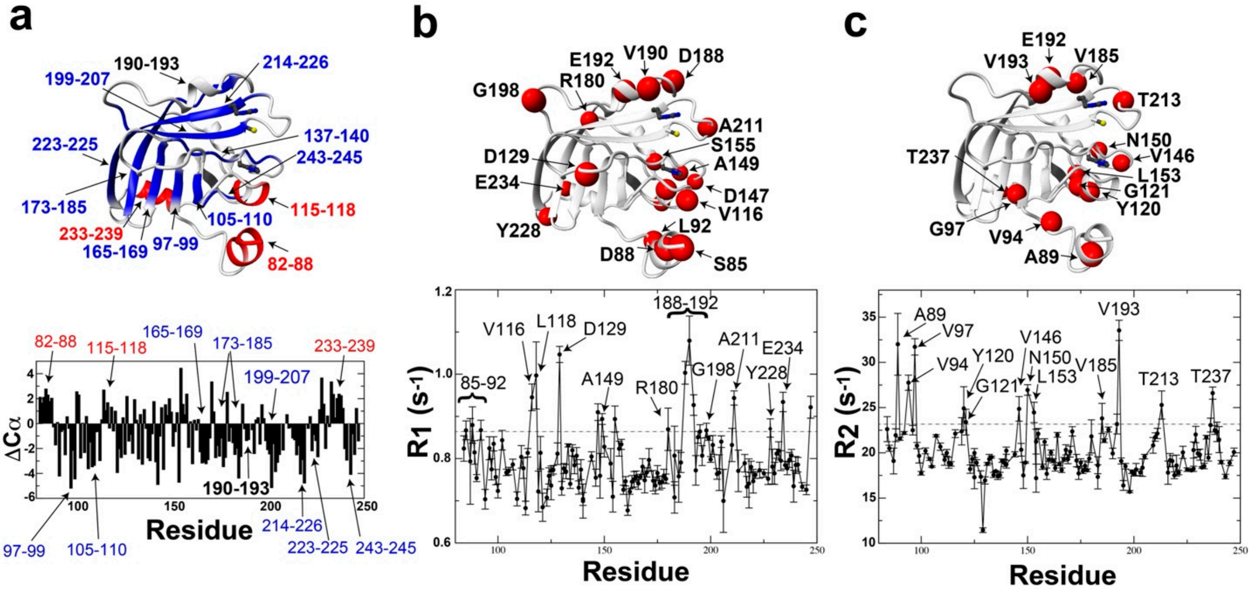

Figure S5. Cα propensities largely agree with the predicted model of *S. pneumoniae* SrtA. (**a**) The differences of the observed backbone Cα resonances relative to that of the unstructured resonance (ΔCα). CSI predicted secondary structure are as follows: Predicted α-helical regions are 82-88, 115-118, and 233-239. Predicted β-strands are 97-99, 105-110, 137-140, 142-144, 165-169, 173-185, 199-207, 214-226, and 243-245. Spectra were collected at 600 MHz at 20 °C. (**b**) R_1 relaxation rates plotted as a function of residue number (bottom) and mapped onto the AlphaFold model (top). The dashed line indicates the average R_1 value (0.79 s^{-1}), while the shaded region (or upper dashed line, depending on the figure) corresponds to ±1 standard deviation (0.07 s^{-1}). Residues exhibiting elevated R_1 values are highlighted in

red on the structure. R_1 data were collected at 900 MHz and 20 °C. (**c**) R_2 relaxation rates plotted as a function of residue number (bottom) and mapped onto the AlphaFold model (top). The dashed line indicates the average R_2 value (20.2 s^{-1}), while the shaded region (or upper dashed line) corresponds to ±1 standard deviation (3.0 s^{-1}). Residues exhibiting elevated R_2 values are highlighted in red on the structure. The β6/β7 loop contains several residues with R_1 and R_2 values exceeding one standard deviation above the protein average, consistent with enhanced backbone dynamics and conformational exchange within this active-site region. R_2 data were collected at 900 MHz and 20 °C.

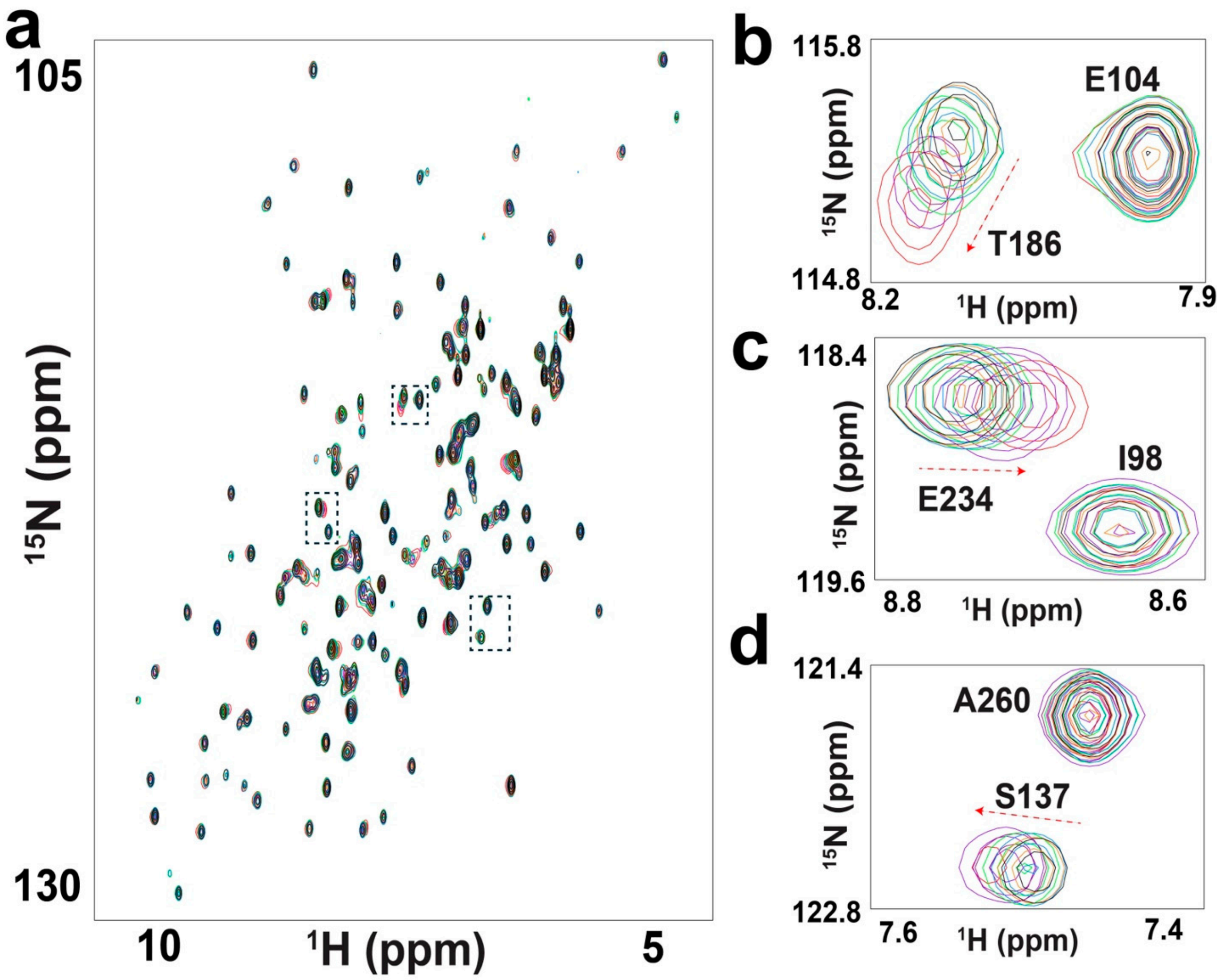

Figure S6. NMR titration of *S. pneumoniae* SrtA with Ca^{2+}. **(a)** Overlay of the ^{15}N-HSQC spectra of *S. pneumoniae* SrtA collected during titration with increasing concentrations of Ca^{2+} (0–50 mM). While most resonances remain unchanged, a subset exhibits progressive chemical shift perturbations, indicating weak Ca^{2+} binding. **(b–d)** Representative blow-ups of dashed boxes in (**a**) illustrating specific regions with induced CSPs.

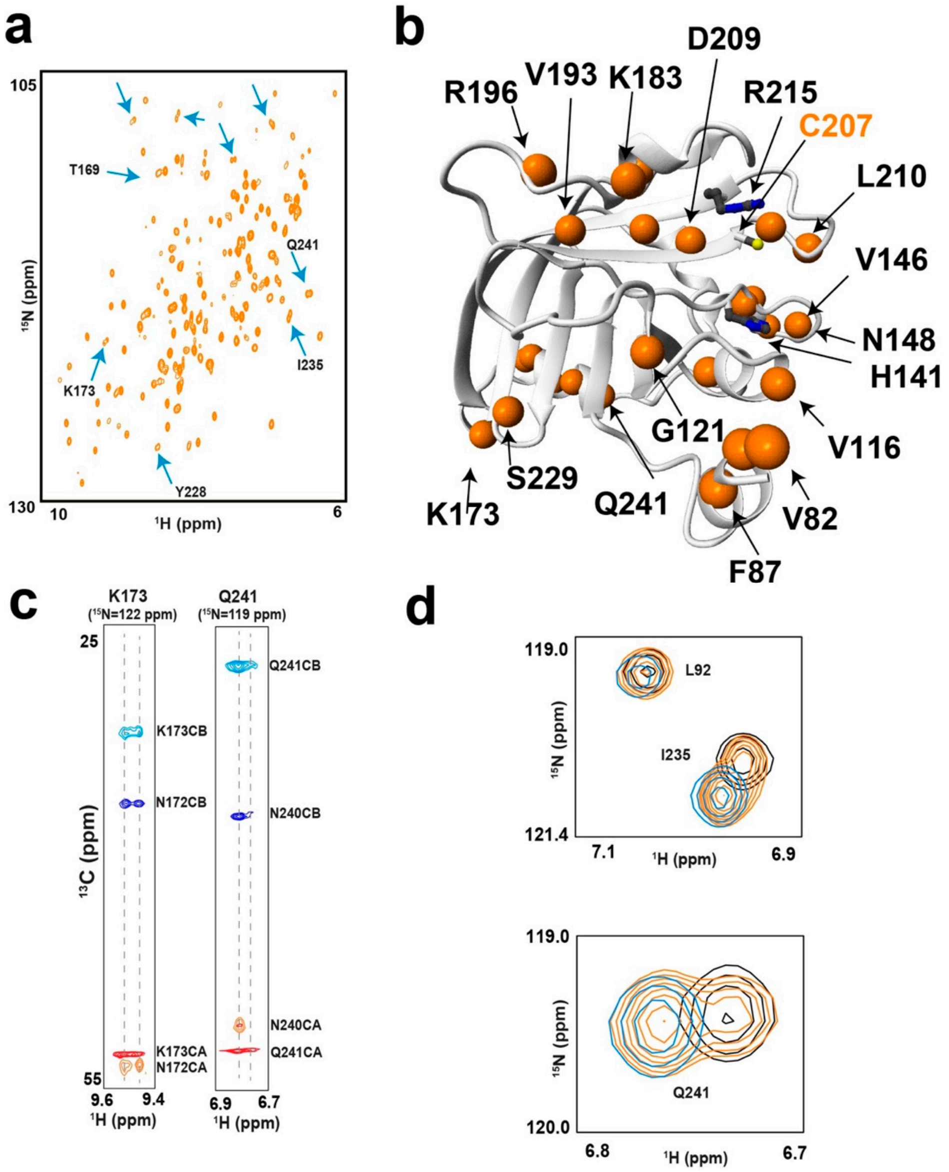

Figure S7. *S. pneumoniae* SrtA binds Ni. (**a**) ^{15}N-HSQC spectrum of the *S. pneumoniae* SrtA monomer without EDTA treatment. Arrows delineate amides with multiple resonances (blue arrows). (**b**) Amides exhibiting multiple resonances are mapped onto the Alpha Fold predicted structure of the *S. pneumoniae* SrtA monomer. (**c**) Slices comprising assigned resonance for K173 and Q241 from a 3D HNCA (red), HNcoCA (orange), HNcaCB (sky blue), and HNcocaCB (navy blue). (**d**) Selected regions of ^{15}N-HSQCs highlighting several amides with duplicate resonances before EDTA treatment (orange), after EDTA treatment (black), and after titration with 1 mM Ni^{2+} (sky blue).

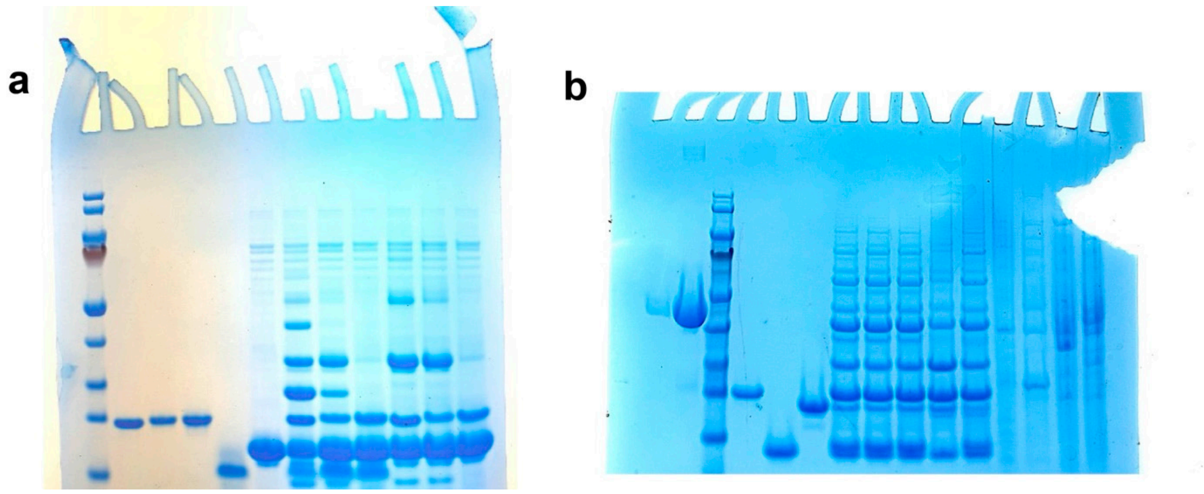

Figure S8. Full, uncropped SDS–PAGE gels corresponding to figures in the main text. (a) Full SDS–PAGE gel corresponding to Figure 3c. (b) Full SDS–PAGE gel corresponding to Figure 4g.
